# Mental health and the workplace: issues for developing countries

**DOI:** 10.1186/1752-4458-3-4

**Published:** 2009-02-20

**Authors:** Prem Chopra

**Affiliations:** 1 Centre for International Mental Health, School of Population Health, The University of Melbourne, Level 5, 207 Bouverie Street, Carlton, Vic 3053, Australia

## Abstract

The capacity to work productively is a key component of health and emotional well-being. Common Mental Disorders (CMDs) are associated with reduced workplace productivity. It is anticipated that this impact is greatest in developing countries. Furthermore, workplace stress is associated with a significant adverse impact on emotional wellbeing and is linked with an increased risk of CMDs. This review will elaborate on the relationship between workplace environment and psychiatric morbidity. The evidence for mental health promotion and intervention studies will be discussed. A case will be developed to advocate for workplace reform and research to improve mental health in workplaces in developing countries in order to improve the wellbeing of employees and workplace productivity.

## Introduction

A key component of the World Health Organisation (WHO) definition of health is the notion of the capacity to participate in community life, rather than the traditional narrower view of health as the absence of disease [1]. According to this definition, health refers to "a state of wellbeing in which the individual...is able to work productively and fruitfully, and is able to make a contribution to his or her community" [1]. Mental health encompasses the individual's capacity to cope with internal needs as well as external needs, such as roles within employment [2]. Existing studies have predominantly focused on the complex inter-relationship between mental health and work productivity in developed countries. Yet according to the WHO, about 75% of the world's labour force is based in developing countries [3]. There is a relative lack of research focusing on this relationship within workplace settings in developing countries.

Mental illnesses, and in particular Common Mental Disorders (CMDs) such as depression and anxiety, are among the most frequent causes of occupational disability [4]. The burden of CMDs is under-recognised in developing countries, despite strong evidence regarding its social impact [5]. Depression is expected to be the second most common disorder across the world behind ischaemic heart disease by 2020 and is expected to account for 15% of the total disease burden [5]. Despite this, several population-based studies in developed countries have demonstrated that CMDs are under-recognised and under-treated. According to the Australian National Survey of Mental Health and Wellbeing for example, only 35% of people identified with mental illness sought treatment [6]. Furthermore the 12-month prevalence of anxiety disorders was 9.7% and depressive disorders was 5.8%, yet only 28% and 40% respectively of individuals sought treatment [6]. Similarly, according to the New Zealand Mental Health Survey, 58% of those with serious disorders and only 36.5% of those with moderate disorders sought treatment in the preceding 12 months [7].

The economic burden of depression alone is substantial [4]. Depression is often chronic and hence may result in enduring disability [4]. Greenberg *et al *(1996) estimated that in 1990, depression cost the US economy US$53 billion annually, of which US$33 billion was a consequence of reduction in work productivity [8]. A follow-up study by the same author in 2000 indicated that although the rate of treatment of depression increased, its economic burden rose only slightly, allowing for inflation, to US$83 billion, suggesting that the economic burden of depression has been relatively stable [9]. It is poignant that in the intervening period there has been relatively little attention given to the concept of the global impact of CMDs with respect to loss in workplace productivity.

Workplace factors may precipitate illness as well as perpetuate disability associated with mental illness. There is good evidence that certain kinds of workplace stress are associated with a higher risk of psychiatric morbidity [10]. It could be expected that the incidence of such workplace stress is higher in developing countries. Thus far, social attention has focused primarily on the impact of harsh working environments on people's human rights, rather than their emotional wellbeing specifically.

This selected review of the recent literature will focus on the current understanding of the relationship between CMDs and work productivity, and also the manner in which workplace environment may adversely impact on mental health. A case will then be presented calling for further attention specifically in developing countries, including research with the agenda of understanding this relationship in order to facilitate the development of effective interventions in the workplace setting.

## Workplace conditions

Most research in workplace mental health has been based in developed countries and hence has focused on the employment conditions defined as fair employment, which refers to employment in which there are clear agreements regarding employer-employee relationships [3]. However there are a variety of employment conditions with more unstable workplace environments, as described in the WHO Employment Conditions Knowledge Network (EMCONET) Report by Benach *et al *(2008) [3]. These include precarious employment, defined as temporary work contracts that offer reduced social security and stability; informal employment, which refers to non-regulated arrangements between employers and employees and represents the most prevalent working condition in developing countries; child labour, particularly the employment of children under the age of 12; and slavery, defined as employment in which individuals are forced to work as a result of being subjected to mental or physical abuse with no relationship with their employer other than as a "commodity" [3].

Specific studies investigating the association between workplace environment and CMDs have been biased towards developed countries and workplaces characterised by fair employment working conditions. It can be expected that the prevalence of psychiatric morbidity will be substantially higher amongst individuals in more stressful working conditions. Furthermore the plight of the unemployed varies significantly between countries and is heavily influenced by the availability of a welfare system, in the absence of which the burden may be borne by working family members, thus adding to the already significant burden [3].

There are various environmental factors that clearly have an influence on the health of workers. At a broad systemic level these include the political environment, policies that govern the labour market, access to basic services including health services and stability of social and family networks [3]. Fairness of workplace processes includes organisational justice, which refers to the fairness of workplace procedures [11]. Low organisational justice has been linked with an increased risk of CMDs [11]. When considering the individual worker, there are hence a myriad of factors that interact and may influence the impact of workplace stress. It may be artificial to separate workplace stress from general environmental stress in many developing countries. The economic status of the nation, living conditions, access to adequate housing and access to recreational pursuits, may all have an indirect impact on the workplace environment [12]. Poverty may also increase the likelihood of other illnesses, which further increase the vulnerability of workers in developing countries [3].

## Globalisation and workplace mental health

As a result of the liberalisation of trade and the exchange of goods and services between countries, globalisation has had a significant impact on social equity [3]. Globalisation has led to a widening gap between the rich and the poor, and workers in developing countries in particular have been marginalised [3]. Furthermore, changes in the nature of work as a result of globalisation have resulted in additional demands on individuals with regards to skills and training, creating additional barriers to employment for those vulnerable to CMDs [3].

Globalisation has created an enormous drive to keep labour costs low [3]. This has led to the exploitation of employees who are not rewarded financially and are often victims of cost-cutting by their employers, leading to them working in hazardous conditions [3]. In many developing countries, occupational health is not a significant government priority and hence the beneficiaries of globalisation are able to continue to exploit these vulnerable workers [3].

The processes of modernisation and industrialisation that has accompanied globalisation have led to a maldistribution of poverty and income levels [3]. In particular, 89% of workers in sub-Saharan Africa and South Asia earn less than US$2 per day [3]. There is a higher representation of workers in the informal economy in developing countries, which comprises 47% of the workforce, compared with only 15% in developing countries [3].

## Workplace stress

As a consequence of the changing nature of work and the impact of globalisation, workplace stress is an issue of increasing importance in the developing world [12]. Workplace stress has been defined by the WHO as a "pattern of physiological, cognitive and behavioural reactions to some extremely taxing aspects of work content, work organisation and work environment" [12].

There are two key models that have been developed to understand the impact of psychosocial stressors at work. The first is the demand-control model, which characterises jobs according to the level of demand on the employee and the level of control he or she is able to exert [13]. The combination of high demands and low control is described as job-strain and is associated with the highest risk for developing CMDs [13]. Job-strain is inequitably distributed, as workers in lower skill level jobs are most likely to be affected with depression [14]. Furthermore, other adverse health outcomes have been associated with job-strain, including heart disease and musculoskeletal problems, which in turn add to the impact of psychological stress [15].

Second, the effort-reward imbalance model characterises jobs according to the balance between the effort made by the employee and the rewards received, which include financial rewards, esteem, prospects of promotion and job security [13]. Psychological stress is most associated with employment in which the rewards do not match the effort made [13].

In reality, it should be noted that the demand-control and effort-reward imbalances are intertwined and ought to be seen as integrated when considering the adverse impact of workplace conditions and also when considering potential workplace interventions to reduce the risk of CMDs [3].

## The association between CMDs and reduced workplace productivity

The pattern of prevalence of CMDs in the workforce is similar to that found in the general population [11]. Regarding the assessment of the impact of mental illness on work productivity, different measures have been used. These include: loss days, or the number of days during which respondents were unable to do their usual activities; cutback days, or the number of days during which activities were reduced; and extra effort days, or the number of days during which individuals were able to function normally but only with significant effort [11]. The cost of working days lost in the European Union due to stress-related illness is estimated to be on average 3–4% of GDP [16]. Estimates are that in the UK stress in the workplace causes a loss of 6.5 million working days a year [17].

In a review of five studies assessing the prevalence of mental disorders, Sanderson and Andrews (2006) found that depression and anxiety disorders were most commonly reported [11]. The studies reviewed included the National Comorbidity Survey (NCS) from the USA, the Australian National Survey of Mental Health And Well-Being (ANSMHWB), the NEMESIS study in the Netherlands, the Ontario Mental Health Supplement in Canada and the UK Household Survey of Psychiatric Morbidity [11]. Individuals with mental disorders were found to have a greater risk of non-participation in the workforce, although this conclusion is limited by the fact that studies have been conducted in developed countries [11].

The National Comorbidity Survey (NCS) study estimated that 3.6% of workers in the US labour workforce suffer from major depression and 18% of the workforce suffers from some form of mental illness at any point in time [18]. Furthermore, people with depression were found to have an increased likelihood of experiencing comorbid physical disabilities, which may in turn have a negative impact on workplace productivity [18].

The NCS Replication study by Kessler *et al *(2006) assessed the association between mood disorders and impairment in the workforce more specifically [19]. In this study of 3,378 workers in the USA, 6.4% met criteria for major depressive disorder. Work performance was assessed using the WHO Health and Work Performance Questionnaire, incorporating self-report regarding absenteeism and presenteeism [19]. Presenteeism refers to the situation in which an employee attends work but is unable to work at their full capacity as a result of their illness; the impact of this issue has become of increasing concern to employers. It has been postulated that presenteeism may be of particular relevance to people with CMDs, as they may be less likely to report mental illness as a reason for missing work [20]. Depressive disorders were found to have a significant effect on work performance. The authors' projections led to an estimate of 225 million workdays lost productivity per year associated with major depressive disorder across the USA labour workforce [19].

The NEMESIS study reported excess loss days of 28.9% for individuals with affective disorders and 17.6% for those with anxiety disorders [21]. Similar associations were reported in the ANSMHWB report but not the NCS [18,22]. However the NCS did report that all affective and anxiety disorders were associated with significant cutback days, and this pattern was consistent with that reported for both affective disorders and generalised anxiety disorder in Australia, and anxiety disorders alone in Ontario [11].

The Mental Health Economics European Network (MHEEN) Report (2005) confirmed the high prevalence of mental health morbidity in the workplace across the European Union [23]. In Sweden for example, 27% of all cases of long-term sick leave are accounted for by mental health problems [23]. In Austria, although there was a reduction in total days of absenteeism between 1993 and 2002, the proportion of total days of absenteeism that was related to mental health problems increased by 56% [23]. In Germany, there was a significant increase of long-term sickness due to mental illness over a similar time frame [23].

In order to investigate the association between depression severity and job performance, Adler *et al *(2006) followed a cohort of 286 patients identified with major depressive disorder and/or dysthymic disorder and compared them with 93 patients with rheumatoid arthritis and also 193 control subjects [24]. The cohort was followed over 18 months, and at the last time point the depression group had significantly greater deficits in job performance than either the rheumatoid arthritis or the control group. Furthermore, job performance remained static between the 6-month and 18-month intervals, reinforcing the chronic nature of disability that can result from depression [24]. However this study has major limitations with respect to the generalisability of the findings as the enrolled participants were predominantly white and only 7% were employed in labouring jobs [24].

In a cohort study of 6,239 employees, selected at random from three major public corporations in the USA, Druss *et al *(2001) demonstrated similar findings regarding the impact of depression on work performance [20]. This study was more representative of the general population with 43.7% of participants being of non-White racial background. Participants completed surveys regarding health and their satisfaction with health care between 1993 and 1995 [20]. Those who reported depressive symptoms were more likely to be female, were younger, less well educated and were more likely to have comorbid medical problems. This study highlights the association between CMDs and absenteeism. The odds of absenteeism due to health reasons were twice as high for employees with depressive symptoms [20]. More significantly, this study highlighted the impact of presenteeism. Druss *et al *(2001) found a significant association between depressive symptoms and reduced effectiveness at work. In one year of the study the odds of decreased effectiveness at work in people with chronic depressive symptoms was seven times that of people without depressive symptoms [20].

Coworkers and supervisors may also be affected by the impaired performance of individuals with CMDs [25]. Coworkers may need to perform additional work to compensate, and hence there is a "spillover" effect on others in the workplace [25]. This is particularly the case where employees work as part of a team; a stressed group of workers will clearly not function as efficiently, which in turn leads to reduced productivity. Furthermore, mental illness may lead to "spillover" effects on the individual's family members, who may themselves be employed or engaged in other social responsibilities [25].

It is important to acknowledge that the inter-relationship between emotional wellbeing and work productivity is complex. People with CMDs may persist with work yet remain unproductive due to personal reasons, workplace culture and stigma [26]. Workplace culture may also promote the view that CMDs are a sign of individual weakness rather than recognising psychiatric illness as arising from an interaction between the individual and his or her environment and recognising the availability of effective treatments [26,27]. The treated prevalence of CMDs in society in general and the workplace in particular is low [6,19,28,29]. Individual employees may not recognise that they are suffering from anxiety or depression, and may lack motivation to seek assistance [26]. Furthermore, even if the employee recognises that they are suffering, they may be fearful of negative consequences if they overt their condition to their employers [26].

## Workplace stress and mental health

There is a growing evidence base that supports the association between workplace stress and the development of CMDs.

Stress in the workplace may have a pervasive effect on employees, leading to exhaustion, anxiety and depression, and even substance abuse. Repeated changes in the workplace can precipitate additional stress [30]. It is well recognised that stress contributes to high levels of absenteeism in the workplace [31]. Stressors have been defined as a set of circumstances which have an adverse impact on a person's equilibrium [31]. This equilibrium is also influenced by the individual's coping strategies and resources, which are inevitably dependent on the person's environment. Stressors may include various factors such as job insecurity, hazardous working conditions, high workload, the threat of violence, unrealistic deadlines, lack of managerial support and retribution from complaints procedures [12,31]. Other social factors also have an impact on work productivity, including interpersonal relationship difficulties, loss and physical illness [31]. It should also be acknowledged that personality profiles, lower levels of personal resources and lower resilience may also be associated with an increased vulnerability of developing CMDs [13].

Workplace culture is a mediating factor in either reducing or increasing stress. Morale, autonomy and team dynamics can have an effect on workplace stress and subsequently productivity [30]. Bullying, discrimination and abuse of employees are extreme examples of poor workplace culture [30]. On the other hand, investment in creating a positive workplace culture can be viewed as an investment in social capital, which is a resource that all individuals can access [30].

The risk of CMDs is higher in workplaces characterised by a high pace of work and low skill discretion [32]. In general, unskilled workers are reported to have a higher risk of CMDs compared with white collar workers. For female employees in particular, lack of job autonomy and decision-making procedures are risk factors for CMDs [32].

Using the demand-control model, there is evidence that jobs characterised by high demands with respect to workload, time pressure and role conflict increase the risk of psychiatric morbidity [11]. Furthermore, workers with low autonomy and authority are most vulnerable, particularly those who have limited external social support [11]. In a survey of more than 1.000 Victorian workers LaMontagne *et al *(2008) demonstrated a clear correlation between job strain and depression [14]. The population-attributable risk was 13.2% for males and 17.2% for females [14].

An imbalance in the effort-reward paradigm has also been associated with an increased risk of psychiatric morbidity [11]. Tsutsumi and Kawakami (2004) recommend redressing the effort-reward imbalance through encouraging employee control over work scheduling tasks and responsibilities, as well as improving rewards, developing additional reward schemes, supervisor training in the maintenance of a positive relationship with employees, and providing incentives to employees for career development [33].

Several other studies have documented an association between workplace stress, defined in various ways, and depression [13]. The type of employment contract may significantly affect psychiatric morbidity. In particular the British Household Panel Survey found an association between precarious employment and psychiatric morbidity, with a significant longitudinal association demonstrated for men [34]. It could be postulated that work security and lack of reward opportunities in relation to the degree of effort can be a potential source of stress [11,23]. Kawakami *et al *(1990) found in a study of male industrial workers in Japan that jobs associated with high levels of stress had a more than 11-fold relative risk of depression [35]. Virtanen *et al *(2007) used antidepressant prescription as a proxy measure for depression in a study of Finnish workers and found a positive correlation between job-strain and depression [36].

Low social support at work has been shown to be associated with an increased risk of depression. In the NEMESIS study, a high degree of social support was negatively associated with depression, with a relative risk of 0.8 [21]. Other studies have confirmed that low social support, including coworker and supervisor support, are associated with an increased risk of depression [13].

Unique work exposures are of course associated with a higher risk of developing CMDs. For example, Fullerton *et al *(2004) found that rescue workers exposed to physical danger had a relative risk of developing depression of 3.5 compared with the rest of the population [37]. In an interesting study by Berg *et al *(2006) of police officers in Norway, particular factors associated with CMDs were identified that could be considered as common to other occupations [38]. These included job pressure and lack of support. Other factors were identified that were specific to the occupation of police work; frequent work injuries were not surprisingly associated with an increased rate of depressive symptoms [38]. Although post-traumatic stress disorder was not specifically investigated, police reported more depersonalisation in comparison with the general population control group [38].

Zammuner and Galli (2005) noted the impact of emotional labour, the act of expressing emotions that are desirable for the organization, which can place a significant burden on the employee's emotional well-being [39]. This occurs as a result of the stress associated with regulating emotions during interactions in the workplace that may be stressful. Emotional labour was associated with burnout. Whilst this is relevant to other workplace settings, the impact of emotional labour in impoverished workplace settings is likely to be magnified [39].

In a study be Wall *et al *(1997), cited by Munn-Giddings *et al *(2005), 11,637 employees of the UK National Health Service (NHS) were interviewed and a high level of psychological distress was found amongst this cohort [17]. This has significant implications as health service staff are in the position of being professional carers, and their role may be compromised by their own mental well-being. The primary stressors faced by employees of health services include lack of resources and dysfunctional team dynamics, rather than the burden of caring for individual patients [17].

In another study of NHS employees in the UK, Loretto *et al *(2005) demonstrated that there are a wide range of personal, environmental as well as workplace factors which influence the well-being of employees [40]. Conflict between work and non-work activities has a significant adverse impact. Support from management and a sense of autonomy were positively associated with wellbeing whilst high work demands and numerous changes at work had a negative impact [40]. Loretto *et al *(2005) found that work pressure is associated with work-life imbalance which in turn has an adverse impact on psychological health, with an increased likelihood of employees suffering from a diagnosable CMD [40].

There have been relatively few studies that may be more relevant to the majority of workers in developing countries. Using the Hopkins Symptoms Checklist (HSCL-25) in a study of 374 female cleaning personnel in Norway, Gamperiene *et al *(2006) found that 17.5% of all personnel had evidence of a CMD. This figure is more than double the average prevalence of CMDs among working women in Norway of 8.4% [32]. The cleaning profession was chosen by the authors as this occupation is known to be associated with several risk factors for stress in the workplace including low pay, lack of esteem and lack of control over working conditions [32]. Poor satisfaction with leadership and poor satisfaction with co-workers were significantly associated with poor mental health [32]. Interestingly, shift work and job strain were not found to be associated with mental health problems in this study [32]. Cleaning staff who were immigrants were three times more likely to have CMDs compared with staff born in Norway. Also, employees in the 50–59 age bracket had a higher prevalence of mental health problems compared with younger employees as well as older employees approaching retirement [32].

It is interesting to note that despite evidence for this association between workplace stress and CMDs, this is usually not sufficient for affected employees to receive compensation [41]. This is due to several factors, including the view that the association may not be proven independent of other stressors [41]. Compensation courts often view depression as a condition that cannot be proven because it does not have any objective signs. Furthermore, compensation courts are wary of potential malingerers [41]. LaMontagne *et al *(2008) also noted that depression associated with job strain is most probably under-recognised, as there are fewer numbers of individuals seeking compensation as a result of job strain [14].

## Workplace mental health interventions

The workplace has been increasingly identified as an appropriate setting for primary care interventions to improve health and also hence in turn improve workplace productivity [15]. Gains from investment in the wellbeing of employees goes beyond financial ones. Greater wellbeing may also lead to improved commitment by employees, reduced labour turnover, quality of goods and services as well as innovation [30]. From a positive stance, intervention in the workplace may have a positive impact on the quality of life of employees and hence improve both economic and social sustainability [30].

Whilst significant progress in the field of health promotion has been made in workplaces in the developed world, the focus has been on stress in general and the identification of individuals with CMDs has not been a specific focus [30]. Although it is common sense that dysfunctional work environments can contribute to the onset of CMDs, particularly in vulnerable individuals, there is a dearth of data regarding the potential impact of workplace stress management programs on the incidence of CMDs [41]. Given the recognised impact of CMDs on productivity, it is surprising that there has not been as yet widespread investment in enhanced assessment and treatment programs in the workplace [42,16].

It is interesting to note that in developed countries, mental health promotion in the workforce has been seen in some ways as separate from public health [23]. This has meant that for example in many European countries there has been a lack of a coordinated effort to institute mental health promotion and intervention programmes in the workplace [23]. WHO have called for an integrated approach to the promotion of mental health in Europe, across communities, educational settings and workplaces [23]. Specifically, there is a need for workplaces to be modified to be conducive to good mental health, including changes to working hours and patterns, exercise and supportive management, as well as providing specific attention to mental health in occupational health and safety programmes [23].

Empirically, it is conceivable that workplace productivity can be improved if CMDs are identified and treated. On the basis of a clinical trial of people with chronic depression, Berndt *et al *(1997) demonstrated an inverse relationship between severity of depression and work performance [43]. Furthermore, treatment improves work performance rapidly with approximately two-thirds of the improvement occurring during the first few weeks [43]. The improvement was greatest with those individuals with the least severity of depression at baseline, supporting the notion that a population-based approach may lead to a greater level of improvement in workplace productivity, rather than exclusively focusing on a clinical subsample of more impaired individuals [43].

The argument for intervention programs that are based in the workplace is further strengthened by population epidemiological studies. As described by Andrews *et al *(2001), according to a collation of data from the Australian National Survey of Mental Health and Wellbeing and the World Health Report, only a third of individuals with a mental disorder sought treatment [29]. The workplace provides an ideal setting where high-risk individuals may receive treatment. There is evidence that work productivity improves with alleviation of the severity of depression [4,44]. Longitudinal studies have confirmed that treatment for depression is associated with a reduction in absenteeism and improvement in individuals' capacity to maintain employment [44]. In addition, treatment for depression may lead to indirect cost benefits as a result of improved workplace productivity and a reduction in the "spillover" effect on other employees [4].

In a cost-effectiveness study in the US, Zhang *et al *(1999) showed that the cost of treatment for depression was completely offset by savings from loss in productivity due to lost work days alone [28]. Furthermore, estimates of the economic burden of depression do not take account of the indirect costs including the burden experienced by individuals' families and the suffering endured by individuals [28]. Employers bear the cost of reduced productivity, and hence as concluded by Zhang *et al *(1999), employers ought to play an important role in providing employees with necessary assistance [28].

It is evident that symptom remission associated with the natural course and also treatment of CMDs is not sufficient to allow depressed workers to resume full productivity. Specifically tailored interventions and rehabilitation efforts are required [45]. Interventions targeted at addressing barriers in the workplace are also important and it has been demonstrated that improvement in time management, output and physical tasks improve workplace retention as well as productivity of individuals with CMDs [45].

Wang *et al *(2007) conducted a randomised controlled trial involving 604 employees in the USA to investigate the impact of a telephone-based support program for employees identified as having depression [42]. Individuals received telephone support and were encouraged to seek treatment from providers to whom recommendations were given [42]. A structured telephone-based psychotherapy program was also offered to individuals who declined referral to clinicians for face-to-face treatment [42]. The WHO Health and Productivity Questionnaire was used to assess workplace performance [42]. Over a 12-month period the intervention group had significantly lower reports of depression severity, and most importantly from the perspectives of employers, reported a significantly higher level of workplace productivity with respect to hours worked as well as having significantly higher job retention rates [42]. Hence enhanced depression treatment not only improved clinical outcomes but also had a positive impact on workplace productivity [42]. Wang *et al *(2007) suggest that such programs may be considered as social capital investments rather than workplace costs [42].

Munn-Giddings *et al *(2005) investigated a participatory approach to the promotion of well-being in two large NHS health services in the UK. Workshops were run in collaboration with senior managers and employees of these services. This process identified the stress experienced by employees in working with limited resources in a high pressure environment. This powerlessness was mirrored by middle management, despite employees' perception that middle management would not believe their views. The Participatory Action approach engaged staff in the task of generating solutions and developing a strategic plan for the service with ownership by all employees [17].

In a review of studies specifically addressing workplace task-restructuring interventions, Bambra *et al *(2007) confirmed that interventions that increase demand or decrease control have an adverse impact on the psychological health of employees [15]. Hence interventions, including job enrichment and enlargement, teamworking and the development of autonomous work groups, that enhance job control may reduce job-strain and hence may have a positive impact on the health of employees [15].

In addition to mental health promotion in the workplace, specific interventions to assist people with CMDs ought to be delivered in a systematic manner. The case for widespread interventions across the workforce is strengthened by findings from the study by LaMontagne *et al *(2008), which identified that depression attributable to job-strain is underestimated by compensation claim statistics in Victoria, Australia, by approximately 30-fold [14]. Primary care interventions ought to include education and provision of appropriate treatment options [46]. Vocational rehabilitation for individuals with CMDs also has a role in improving personal coping skills and providing improvements to work tasks in order to enable individuals to be productive in their work [46].

## Workplace mental health interventions in the developing world

Whilst health promotion in workplace settings has received attention in the developed world, the focus on mental health promotion has been on stress in general and the identification and treatment of individuals with CMDs has not been a specific focus [30]. Employers in developing countries may be more likely to enforce attendance of employees when unwell; hence it could be expected that higher rates of mental illness and a greater level of presenteeism may contribute to even lower productivity [30].

In developed countries, the welfare system provides a public "safety net", as a result of which the burden of unemployment is shared by the government [25]. In the absence of a welfare system that may protect individuals who are unable to work as a result of their mental illness, workers in developing countries are likely to continue to work despite their disability [25]. The impact on workplace productivity in developing countries is hence magnified, and goes beyond the direct costs as a result of impairment in the workplace. Given the evidence for the effectiveness of workplace interventions, workplace interventions in developing countries ought to be seen as an investment in social capital [42]. It could hence be argued that the workplace provides a critical setting for health promotion, screening of individuals with CMDs as well as a focal point for the provision of interventions and identifying individuals who would benefit from referral to mental health professionals for further management. However advocacy is critical to improve working conditions in impoverished settings in order to prevent psychiatric morbidity and to improve the quality of life of workers.

The economic benefits that may arise from improving workplace conditions and reducing the burden of mental illness in the workplace are substantial, and it is highly likely that demonstration of the cost effectiveness of such programs to employers in developing countries would improve their uptake [23].

It is interesting to note that the focus of employers in developed countries has been on the retention of the ageing workforce, and creating incentives for individuals to remain in employment, rather than alternatives such as taking early retirement. In the absence of adequate welfare systems and the lack of opportunities for life beyond retirement age, the focus of employers in developing countries is clearly a different matter [47]. Advocacy for improving workplace mental health must focus on fundamental changes in labour market reform and further control over informal working conditions that are so prevalent in developing countries [3]. Such reform may in turn lead to a happier and more productive workforce [3].

Workplace reform interventions must be empowering and involve multidisciplinary and multisectoral cooperation [12]. There is a need for collaboration between the primary health care sector and employers in particular [25]. Incentive systems provided by governments to employers to improve the effort-reward imbalance may reduce the psychological stress experienced by employees [25]. In addition, collaboration between government departments and other stakeholders responsible for health, welfare and the labour workforce is necessary to reduce inefficiencies by pooling resources to coordinate mental health promotion activities in the workforce and the promotion of work-life balance [12,25]. As described by LaMontagne *et al *(2008), improved living conditions, improved access to primary health care and stronger communities may reduce the impact of psychosocial stress in the workplace [14].

## Conclusion

The interaction between mental illness and workplace environment is complex and multifaceted. CMDs have a negative impact on workplace productivity and adverse workplace environments are associated with a higher prevalence of CMDs. Studies thus far have focused on mental health promotion and interventions to treat CMDs in the workplace, primarily in developed countries. However by contrast there are stark differences in workplace environment and standards in the developing world. In the current era of globalisation, greater attention is required to address the imbalance between workplace standards in the developed and developing worlds. Advocacy and research in mental health promotion and interventions to address CMDs in the workplace setting in developing countries is an urgent priority.

## Abbreviations

CMDs: Common Mental Disorders.

## Competing interests

The author declares that they have no competing interests.
